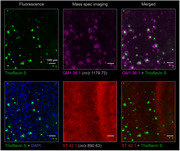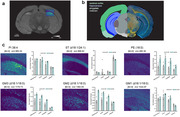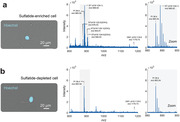# Mapping lipids associated with Aβ plaques in a mouse model of Alzheimer’s disease via multiscale mass spectrometry imaging

**DOI:** 10.1002/alz.095788

**Published:** 2025-01-09

**Authors:** Timothy Trinklein, Stanislav S. Rubakhin, Marisa Asadian, K.R. Sabitha, Orly Lazarov, Fan Lam, Jonathan V Sweedler

**Affiliations:** ^1^ University of Illinois Urbana Champaign, Urbana Champaign, IL USA; ^2^ University of Illinois Chicago, Chicago, IL USA

## Abstract

**Background:**

Increasing evidence shows that many lipids play important roles in the pathogenesis of Alzheimer’s disease (AD), including Aβ plaque formation. Of note, the greatest genetic risk of late onset AD, apolipoprotien E4 (APOE4), plays a major role in lipid transport. However, the profile of lipids that play a role in AD is poorly understood. Mass spectrometry imaging (MSI) is a tool of choice for quantifying and spatially mapping lipids in biological samples. Here, we apply MSI at the tissue‐ and single cell‐level to profile lipids in the brains of 5xFAD and wild type mice to identify lipids associated with Aβ plaques.

**Method:**

Female 5xFAD mice at 5 months of age (*n* = 3) and age‐ and sex‐matched wild type (*n* = 3) were used. After sacrifice, brains were removed and a coronal slice was frozen and used to obtain 12 µm tissue sections. Fresh tissue was dissected, dissociated into single cells, and dispersed onto glass slides. Using matrix assisted laser desorption ionization (MALDI) MSI, we imaged tissue slices (*n* = 12, two per animal) and over 10,000 individual cells. After MSI, sections were stained with Thioflavin S and imaged by epifluorescence microscopy. Statistically significant lipids between classes were discovered by the Wilcoxen rank‐sum test with Benjamini‐Hochberg correction.

**Result:**

Co‐registration of mass spectrometry imaging data with histological staining allowed us to identify a suite of plaque‐colocalized and depleted lipids (Fig. 1). We found that many lipid classes including monogangliosides (GM), hexosylceramids (HexCer), phosphatidic acids (PA), phosphatidylethanolamines (PE), phosphatidyleinositol (PI), and lysophosphatidylecholines (LPC) were plaque‐colocalized and in higher abundance in 5xFAD animals relative to controls (Fig. 2). In contrast, sulfatides (ST) were depleted in plaques and in lower abundance (Fig. 2). In total, we identified 20 plaque‐enriched lipids and 5 plaque‐depleted lipids. All 25 lipids were detected in single cells, recapitulating the trends shown in tissue‐level data (Fig. 3).

**Conclusion:**

Our finding revealed accumulation and depletion of lipids in the plaque microenvironment and single cells. Work is ongoing to extend our approach to different ages (to observe disease progression) and other models, such as APOE3/4 knock‐in animals.